# Uveitis–Glaucoma–Hyphaema Syndrome. General review

**DOI:** 10.22336/rjo.2017.3

**Published:** 2017

**Authors:** Mihail Zemba, Georgiana Camburu

**Affiliations:** * Ophthalmology Department, “Dr. Carol Davila” Central Military Emergency University Hospital, Bucharest, Romania

**Keywords:** UGH syndrome, uveitis, glaucoma, hyphema, cystoid macular edema, IOL, anti-VEGF Therapy

## Abstract

Uveitis-Glaucoma-Hyphaema Syndrome (UGH syndrome, or “Ellingson” Syndrome) is a rare condition caused by the mechanical trauma of an intraocular lens malpositioned over adjacent structures (iris, ciliary body, iridocorneal angle), leading to a spectrum of iris transillumination defects, microhyphaemas and pigmentary dispersion, concomitant with elevated intraocular pressure (IOP). UGH Syndrome can also be characterized by chronic inflammation, secondary iris neovascularization, cystoid macular edema (CME). The fundamental step in the pathogenesis of UGH syndrome appears to arise from repetitive mechanical iris trauma by a malpositioned or subluxed IOL. These patients have uncomplicated cataract implants and return for episodes of blurry vision weeks to months after surgery. This may be accompanied by pain, photophobia, erythropsia, anterior uveitis, hyphaema along with raised intraocular pressure. A careful history and examination, as well as appropriate investigations can confirm the diagnostic. Treatment options are IOL Explantation exchange, topical and systemic medication, and cyclophotocoagulation, the placement of a Capsular Tension Ring to redistribute zonular tension and Anti–vascular endothelial growth factor (anti-VEGF) Therapy.

## Background

Uveitis-Glaucoma-Hyphaema Syndrome (UGH syndrome, or “Ellingson” Syndrome) is a rare condition caused by the mechanical trauma of an intraocular lens malpositioned over adjacent structures (iris, ciliary body, iridocorneal angle), leading to a spectrum of iris transillumination defects, microhyphaemas and pigmentary dispersion, concomitant with elevated intraocular pressure (IOP). UGH Syndrome can also be characterized by chronic inflammation, secondary iris neovascularization, and cystoid macular edema (CME) [**1**].

The term UGH Syndrome was originally described by Ellingson in 1977 as a result of excessive lens movement by small lens size or lens dislocation. Poorly manufactured edges, iris-clipped IOLs, or rigid closed looped haptics were also found as promoting factors. Along with the upgrade of the lens design, fabrication, surgical techniques, and the use of posterior chamber IOLs, the incidence of UGH has sharply decreased from a mean of 2.2 to 3% to 0.4 to 1.2% over a one-year period [**2**].

## Epidemiology 

The UGH Syndrome is a triad that commonly occurs in adults, nonetheless it was reported a case of postoperative uveitis-glaucoma-hyphaema syndrome following pediatric cataract surgery [**3**].

Speaking at the ASCRS 2016, Dr. Albert Cheung stated that UGH is a rare, but potentially devastating complication. Dr. Cheung’s 10-years (2005-2015) retrospective chart review is the result of 249 patients who had been referred for evaluation for IOL reposition or exchange, 53 of them (56 eyes) having UGH at presentation [**4**].

Although it was mainly described with rigid anterior chamber lenses, the UGH syndrome has also been described with posterior-chamber and iris-supported lenses. In a retrospective review from Gainesville, University of Florida, 97 patients who had some form of UGH were studied and 54% had an ACIOL, while 34% had an iris-fixed lens. UGH syndrome can occur in cases with PCIOLs, however, it is much less likely due to the added stability provided by the lens capsule [**5**].

The same result was found in Hong Kong Eye Hospital’s medical records of the patients who underwent IOL explantation during January 2008 and March 2013. The reasons for lens removal from a total of 98 explanted IOLs included in the study were due to lens malposition (71.4%), isolated uveitis-glaucoma-hyphaema (UGH) syndrome (9.1%), refractive surprise (6.1%), and pseudophakic bullous keratopathy (4.1%), “In-the-bag” IOL malposition associated with intraocular complications during cataract extraction (28.9%) and high myopia (22.2%). Sulcus-fixated 3-piece lenses had the UGH Syndrome as a complication in 7.1% of the cases, whereas Sulcus implantation of a single-piece acrylic (SPA) was involved in all cases [**6**].

## Pathophysiology

As the overall pathogenesis of the UGH syndrome remained unclear for a long period of time, some hypotheses were made. These theories were based on the activation of the innate immunity: cytokine and eicosanoides synthesis, triggered by a mechanical excoriation of the angle or iris, by the haptics or optics, plasma-derived enzyme (especially complement or fibrin) activated by the surface of the IOLs (especially PMMA), adherence of bacteria and leukocytes to the IOL surface, toxicity caused by contaminants on the IOL surface during manufacturing implantation were suggested [**5**].

Ultrasonic biomicroscopy has enabled a better understanding of the mechanism of this disorder. In a review of 20 suspected cases, ultrasonic biomicroscopy showed the haptic in contact with the iris in 75% of the cases, extending to the ciliary body in 35% and to the pars plana in 10% of the cases [**7**]. The lenses have surface imperfections that may render them more capable of traumatizing the tissue and cause symptoms. Vaulting, decentration, and excessive movement of the lens may cause the breakdown of the blood-aqueous barrier. Intermittent contact with the fragile vascular uveal tissue may then lead to chafing, erosion, and pigment dispersion; consequently, with signs of anterior uveitis and recurrent episodes of hyphaema, and raised intraocular pressure. Elevated intraocular pressure is a result of persistent inflammation, pigment dispersion and deposition, or secondary to macrophages containing degraded red blood cells blocking the trabecular meshwork. Further, the patient may develop glaucomatous atrophy and visual field loss.

**Fig. 1:**
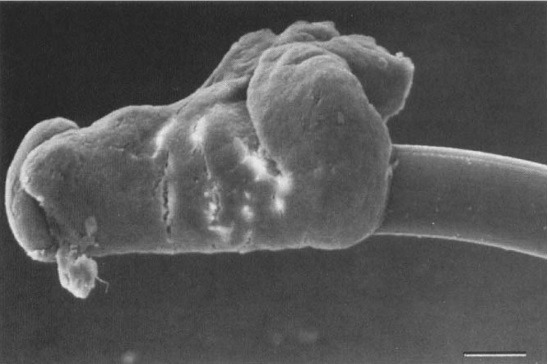
Scanning electron micrograph showing material attached to the tip of a haptic lens. R. H. Y. ASARIA J. F. SALMON Oxford

Previous studies suggested that the low-grade chronic inflammation was the effect of a foreign-body reaction to the lens material or to a toxic compound on the lens surface. Lately it has been proved that cause is the avirulent organisms that are sequestrated within the lens haptic. Performing the scanning electron microscopy of cellular findings on explanted IOLs in patients with UGH, revealed coccoid-like structures on the haptic surface. Moreover, transmission electron microscopy made the spherical structures turn into melanosomes, possibly derived from the damaged pigment epithelial cells or stromal melanocyte in consequence of recurrent contact of the haptic against the anterior or posterior part of the iris [**8**].

**Fig. 2:**
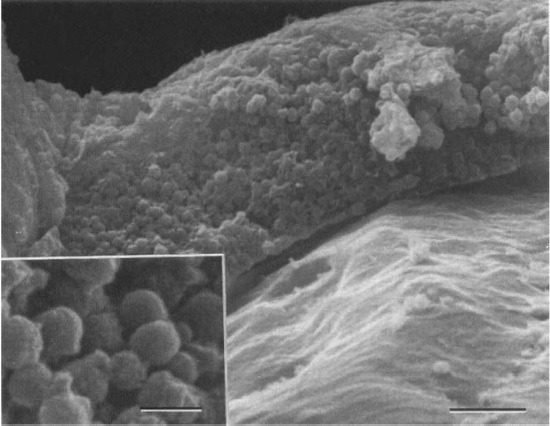
Details of the surface of the material attached to the haptic tip showing densely packed coccoid-like structures. R. H. Y. ASARIA , J. F. SALMON, Oxford - Electron microscopy findings on an intraocular lens in the uveitis, glaucoma, hyphaema syndrome

The fundamental step in the pathogenesis of the UGH syndrome appears to arise from repetitive mechanical iris trauma by a malpositioned or subluxed IOL.

Cheng et al., Jacobs et al., and Miller and Doane determined the movements of IOLs by using high-speed-imaging, for short-distance eye movements. Previously, position-determining investigations of IOLs in various positions or during accommodation were made, but not during fast eye movements.

A Pilot Study was conducted in the *Centre for Ophthalmology, University Eye Hospital, Eberhard Karls University of Tübingen*, Germany to measure this condition in a more realistic manner, and aimed to evaluate the kinetic influences, in fast direction changes and at lateral end points, with a digital high-speed camera along with digital morphometric software for dynamic measurements of phakic intraocular lens movements. The selected “peak lens deviation” images that were analyzed in the study revealed a low-lying lens position relative to the pupil center in nine of ten eyes. Possible explanations for this result were described as implantation position, looseness of the iris or fixation points, shifting haptic phenomenon, and gravity forces “pulling” the lenses. Other studies also showed decentrations of the ICIOL models. Menezo et al. asserted decentrations of up to 1 mm, and, at the same time, Pérez-Santonja et al. decentrations were greater than 0.5 mm in 14 (43%) of 32 eyes [**9**].

**Fig. 3:**
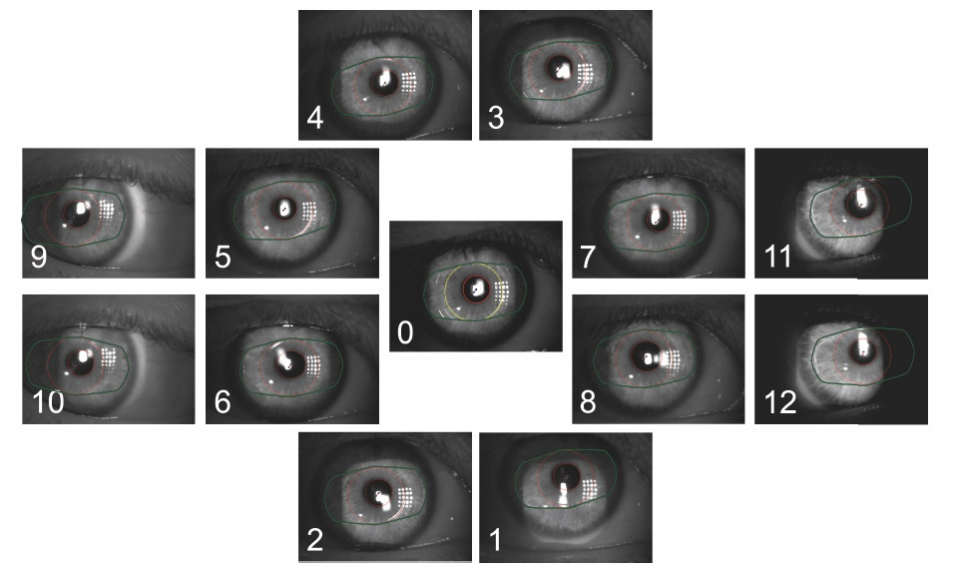
Images of different positions with overlays of the pupil shape and lens positions. Leitritz, Ziemssen-Tübingen, Germany, Using a slit lamp-mounted digital high-speed camera for the dynamic observation of phakic lenses during eye movements: a pilot

UGH is most commonly caused by anterior chamber intraocular lenses, but can occur from any type of pseudophakic lens. Angle supported anterior chamber implants have been in use since the 1950’s for the correction of aphakia. 75% of the patients with this type of implant manifested symptoms that can relate to the UGH syndrome. The lens direct contact with the iridocorneal angle structures produces continuous mechanical damage and pigment dispersion. The liberated pigment occludes the trabecular meshwork and rise IOP. Since ACIOL were promoted for their cosmetic use, there have been a growing number of cases with UGH [**10**].

The frequency of UGH syndrome as a complication of posterior chamber lens implantation is very low. Although Percival reported a case with an implantation of a Rayner-Pearce tripod lens, Van Liefferinge described two cases with the implantation of an Anis-type lens. In most of these cases, the implanted lenses were modern single-piece or 3-piece lenses. In these cases, the unstable sulcus fixation caused mechanical irritation and trauma to the surrounding tissues and vessels. Although electron microscopy did not show any deterioration of the haptic material, the decreased size of the lens might have caused the rotation. Cases of UGH Syndrome were described even with an adequate positioning in the bag for a single block IOL with square edged haptics in specific situations, such as zonular latitude and plateau iris [**11**].

## Presentation

UGH is a complication that can occur after post-op cataract surgery. These patients have uncomplicated cataract implants and return for episodes of blurry vision weeks to months after surgery. This may be accompanied by pain, photophobia, erythropsia, red eyes, anterior uveitis along with raised intraocular pressure. There is never a complete loss of light perception. Regarding the slit lamp examination, the presence of microscopic hyphaema can conduct the diagnose. Occasionally, the intraocular bleed is sufficient to produce a macroscopic hyphaema, which is visible without a slit-lamp. Neovascularization of the iris or corneal edema (apposition of the prolapsed intraocular lens over the corneal endothelium) can also be included as other features. Gonioscopy can be valuable because it may disclose blood in the trabecular meshwork between attacks [**12**].

A careful history and examination, as well as appropriate investigations can confirm the diagnostic.

Variations of UGH syndrome include UGH Plus and IPUGH (Incomplete Posterior UGH). IPUGH is defined as bleeding into the posterior chamber with/ without glaucoma and no uveitis. UGH Plus is defined as a UGH syndrome plus a vitreous hemorrhage and occurs more frequently with anterior chamber lenses with iris support, and it is also described with posterior chamber lenses positioned in the sulcus or in the capsular bag. The loss of anterior hyaloids integrity (spontaneous, degenerative or after surgery) enables communication between the aqueous humor and the vitreous, allowing the passage of blood and giving rise to the possibility of simultaneous bleeding in both chambers. The coexistence of VH should not delay the diagnose [**13**].

**Fig. 4:**
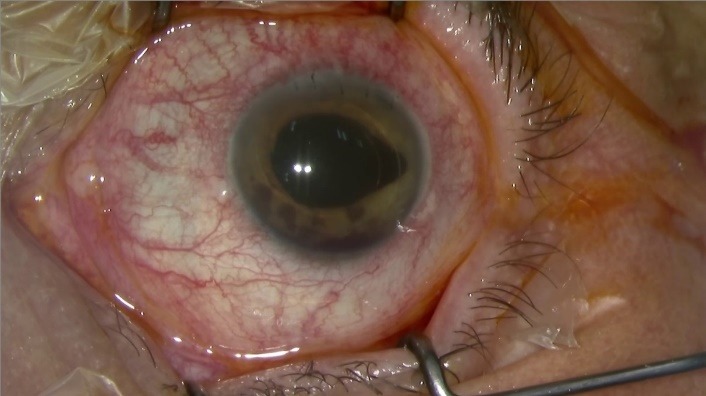
Uveitis-Glaucoma-Hyphaema Syndrome, Zemba M, Camburu G, Ophthalmology Department, “Dr. Carol Davila” Central Military Emergency University Hospital, Bucharest, Romania

## Investigations

OCT-SD and/ or BMC-US supports the diagnose by showing the IOL position and its relationship with the surrounding ocular structures. Ultrasound biomicroscopy (UBM) is often used in the diagnosis of UGH syndrome to visualize the malpositioned IOLs, to confirm the haptics position and their contact with the uveal tissue. In addition, ocular coherence tomography (OCT) can aid in the guidance of diagnosing CME [**13**].

**Differential Diagnosis**

Trauma

Vascular abnormalities

Rubeosis iridis

Ocular ischemic syndrome

Diabetes mellitus

Retinal artery occlusion

Retinal vein occlusion

Swan Syndrome (NV wound)

Juvenile Xanthogranuloma

Iris Varices

Vascular tufts

Hereditary hemorrhagic telangiectasia

Inflammation (Iritis)

Fuchs heterochromic iridocyclitis

Herpes simplex

Herpes zoster 

Iatrogenic Causes

Intraocular surgery

Laser trabeculoplasty

Iridotomy 

Neoplasm 

Retinoblastoma

Melanoma

Iris hemangiomas

Systemic Disorders

Sickle cell trait or disease

Coagulation disorders

Anticoagulation medications

**Complications**

Pseudophakic bullous keratopathy (PBK)

Corneal straining

Chronic inflammation

Vitreous hemorrhage

Glaucomatous nerve damage

Cystoid macular edema

## Treatment 

In patients with UGH syndrome, topical and systemic medication (Corticosteroids along with IOP lowering medication) reduce the intraocular pressure, control the anterior inflammation and bring symptomatic relief in the short term [**14**]. Parasympathomimetics should be avoided because of its miotic effect and increase in the mechanical chaffing to the iris. The management of patients with hyphaema should be limited activity, head elevation, and cycloplegics for ciliary spasm [**15**].

IOL exchange should be performed if the vision is reduced, or raised intraocular pressure and inflammation cannot be controlled or progressive glaucomatous atrophy is demonstrated.

Out of 259 surveys, the IOL Explantation indications given at ASCRS & ESCRS 2000 were the following: ACIOL & Iris fixated lenses (PBK with corneal decompensation, UGH syndrome with CME), Older PCIOL & PMMA PCIOL (Decentration, Corneal edema and inflammation), Foldable 3pc monofocal silicone PCIOL (40% incorrect lens power 32% decentration, 9% damaged IOL during insertion), Foldable 3 on acrylic PCIOL (39% incorrect lens power, 24% optical aberration, 15% decentration), Foldable plate-haptic silicone PCIOL (> 50% decentration, 22% incorrect lens power, 18% damaged IOL during insertion), Foldable 3 on multifocal silicone PCIOL (89% optical aberration, 11% others). Besides the IOL Explantation exchange, topical and systemic medication, and cyclophotocoagulation, new treatment options can be the placement of a Capsular Tension Ring to redistribute zonular tension and Anti–vascular endothelial growth factor (anti-VEGF) Therapy. Intravitreal and intracameral bevacizumab have demonstrated to induce the regression of iris neovascularization and the inflammation in uveitic macular edema. An increase in acute and sustained intraocular pressure can be a potential complication of this therapy. To avoid an immediate increase in the intraocular pressure, an anterior chamber paracentesis can be performed along with the intracameral injections. Corneal endothelium has demonstrated a good tolerance to bevacizumab for doses of up to 2.5 mg, but repeated intracameral injections may cause endothelial cell loss. In defiance of the potential side effect, serial intracameral bevacizumab could offer a temporizing or long-term option to high-risk IOL manipulation cases [**16**].

## Conclusions

In the 1950s, rigid ACIOLs were used in cataract extractions, Bullous keratopathy, inflammation, and cystoid macular edema were very common complications. Today, PMMA, acrylate, and silicone lenses are often used and many choices exist for implantable lenses, from toric lenses to multifocal and accommodating IOLs.

In conclusion, it is important to know the severe effects, signs, and management course for UGH syndrome. UGH syndrome is a severe complication of cataract extraction and a cause for blurry vision weeks to months after surgery. The hyphaema may obscure the doctor’s view of the anterior chamber, posterior chamber, and IOL, a B-scan ultrasound being useful. Lately, the explantation of the IOL has been necessary.

Prior to the operation, all the factors that could affect the difficulty of the surgery should be noticed: zonular laxity, small pupil, medications (anticoagulants), patient’s ability to lay flat, co-existing conditions. These characteristics could become risk factors that cause the decreased vision post-op.

There are advantages and disadvantages in selecting the lens design. Single piece acrylic lenses allow the use of smaller incisions while large haptics lens do not provide the best fit in the sulcus. The 3-piece lens design will fit well in the sulcus but they require a large incision. Overall, it is important to recognize the pros/ cons of the lens designs and adapt that to the patient [**17**].
